# Sagittal Plane Deformities in Children with SMA2 following Posterior Spinal Instrumentation

**DOI:** 10.3390/children8080703

**Published:** 2021-08-16

**Authors:** Matthew A. Halanski, Rewais Hanna, James Bernatz, Max Twedt, Sarah Sund, Karen Patterson, Kenneth J. Noonan, Meredith Schultz, Mary K. Schroth, Mark Sharafinski, Brian P. Hasley

**Affiliations:** 1 Department of Orthopaedic Surgery, University of Nebraska Medical Center, Omaha, NE 68198, USA; mhalanski@childrensomaha.org (M.A.H.); max.twedt@unmc.edu (M.T.); 2 School of Medicine and Public Health, University of Wisconsin-Madison, Madison, WI 53706, USA; rbhanna@wisc.edu (R.H.); JBernatz@uwhealth.org (J.B.); Sund@ortho.wisc.edu (S.S.); pattersonk@pt.wisc.edu (K.P.); Noonan@ortho.wisc.edu (K.J.N.); msharafinski@uwhealth.org (M.S.); 3 Novartis Gene Therapies, 2275 Half Day Road, Suite 200, Bannockburn, IL 60015, USA; mschultz465@avexis.com; 4 Cure SMA, 925 Busse Road, Elk Grove Village, IL 60007, USA; mary@curesma.org

**Keywords:** spinal muscular atrophy, posterior spinal fusion, kyphosis, sagittal plane deformity

## Abstract

This is a retrospective radiographic review to assess post-operative sagittal plane deformities in patients with Spinal Muscular Atrophy type 2 that had been treated with posterior spinal instrumentation. Thirty-two patients with a history of either spinal fusion (N = 20) or growing rods (N = 12) were identified with an average of 7.6 (2.1–16.6) years post-operative follow-up. Forty percent (13/32) of the patients were identified as having obvious “tucked chin” (N = 4), “tipped trunk” (N = 9), or both (N = 3). Sacral incidence was the only parameter that was statistically significant change between pre-operative or immediate post-operative measurements (66.9° vs. 55.2° *p* = 0.03). However, at final follow-up, the post-operative thoracic kyphosis had decreased over time in those that developed a subsequent sagittal deformity (24.2°) whereas it increased in those that did not (44.7°, *p* = 0.008). This decrease in thoracic kyphosis throughout the instrumented levels, resulted in a greater lordotic imbalance (30.4° vs. 5.6°, *p* = 0.001) throughout the instrumented levels in the group that developed the subsequent cervical or pelvic sagittal deformities. In conclusion, sagittal plane deformities commonly develop outside the instrumented levels in children with SMA type 2 following posterior spinal instrumentation and may be the result of lordotic imbalance that occurs through continued anterior growth following posterior instrumentation.

## 1. Introduction

Spinal Muscular Atrophy (SMA) is the most common fatal genetic disease affecting the pediatric population (1 in 6–10,000 live births). Classically, before the widespread use of disease modifying agents, children with this disease experienced progressive weakness and early mortality. SMA is classified into three types based on the onset of disease: type 1 has symptoms starting before 6 months of age, type 2 has onset between 6–18 months of age, and type 3 has onset after 18 months of age [1]. Children with type 1 never sit and without intervention have a life expectancy <2 years, type 2 sit but do not walk and survive into the second decade, and type 3 ambulate and have a life expectancy into adulthood [1]. Respiratory failure is the most frequent cause of death in children with SMA type 1 or 2 [2].

**Figure 1:**
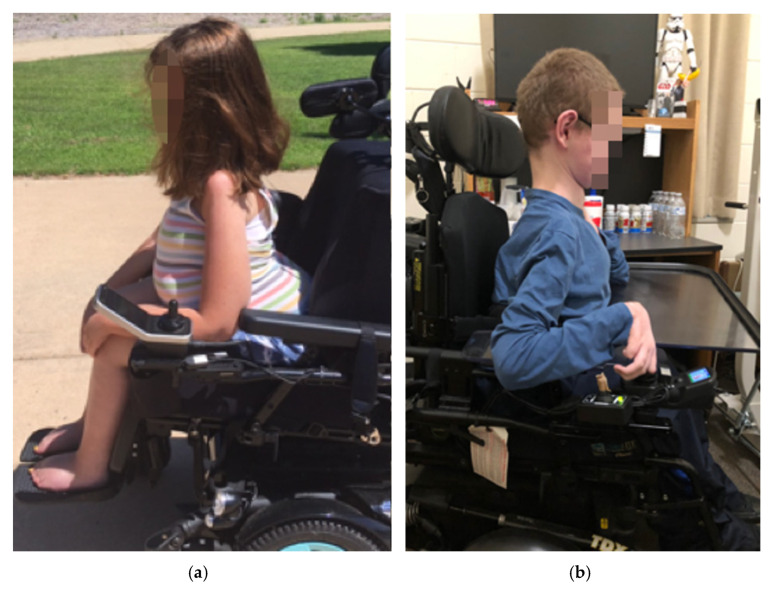
Clinical photos of “trunk tip” (**a**) and “tucked chin” (**b**) deformities. With the “trunk tip” deformity, note the space between the back of the chair and the posterior chest wall.

Our institutional experience in treating severely affected children with SMA (types 1 and 2) [3,4,5,6,7,8,9] with spinal deformities [10,11] has led to clinical observations that a sub-set of children with SMA type 2 (upright wheelchair sitters) developed very characteristic sagittal plane deformities following spinal instrumentation that resulted in either: (1) a “tipped trunk” deformity, in which the entire (fused) and unsupported trunk leans forward causing the abdomen to rest on the anterior thighs in the sitting position, resulting in a very prominent buttock posteriorly that complicates seating support of the lumbar and thoracic spine (Figure 1a), or a (2) a “tucked chin” deformity in which the angle of the jaw appears retracted (Figure 1b). The primary purposes of this study were to (1) screen lateral radiographs to determine the prevalence of these deformities in our post-instrumentation SMA type 2 population (2) use radiographic measurements to objectively characterize the deformities and (3) to set out to determine whether the sagittal deformities were present before, immediately after, or if they developed slowly over time following posterior spinal instrumentation. Additionally, we attempt to identify factors associated with deformity development. Our hypotheses were that these deformities developed slowly over time following spinal instrumentation and that a loss of thoracic kyphosis at the time of instrumentation would contribute to the deformity development.

## 2. Materials and Methods

**Figure 2:**
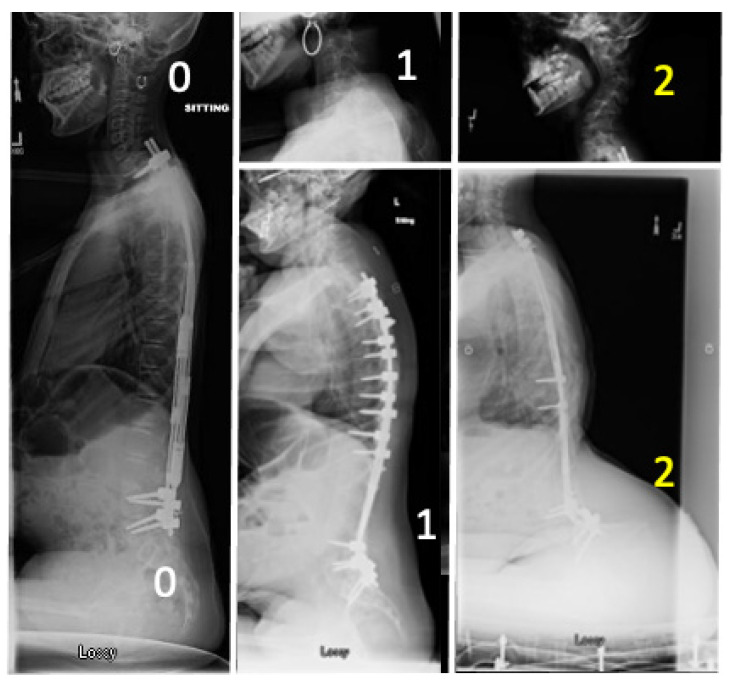
Examples of scoring the latest available radiographs in this study. (**Left**) Normal (0,0) scoring of no tucked chin or trunk tip. (**Middle**) Transitional scores of 1 for slight visual tucked chin (top) and prominent buttock (bottom). (**Right**) Obvious tucked chin (top) and tipped trunk (bottom) scoring a 2 in our system. Only those scoring a 2 (denoted in yellow) were included in our Deformity Cohort, these were compared against those scoring 0 or 1 (white).

A radiographic review of SMA Type 2 patients that had undergone posterior spinal instrumentation (instrumented fusion or growing rod insertion) was performed. As we did not have standardized lateral clinical photographs of every child treated at our institution at each clinical encounter, we used the most recent lateral scoliosis radiograph as a proxy to their physical clinical examination or photographs to identify those patients who had the characteristic sagittal deformities that we have observed in either the cervical spine or trunk. The overall sagittal alignments were graded as 0 (normal), 1 (borderline), 2 (obvious) then classified as cervical (“tucked chin”), pelvic (“tipped trunk”), or both (Figure 2). Patients were then grouped into two cohorts those with or without obvious deformities (grade 0 or 1 vs. grade 2). These radiographs were reviewed and scored by a fellowship trained pediatric orthopedic spinal deformity surgeon (MAH).

**Figure 3:**
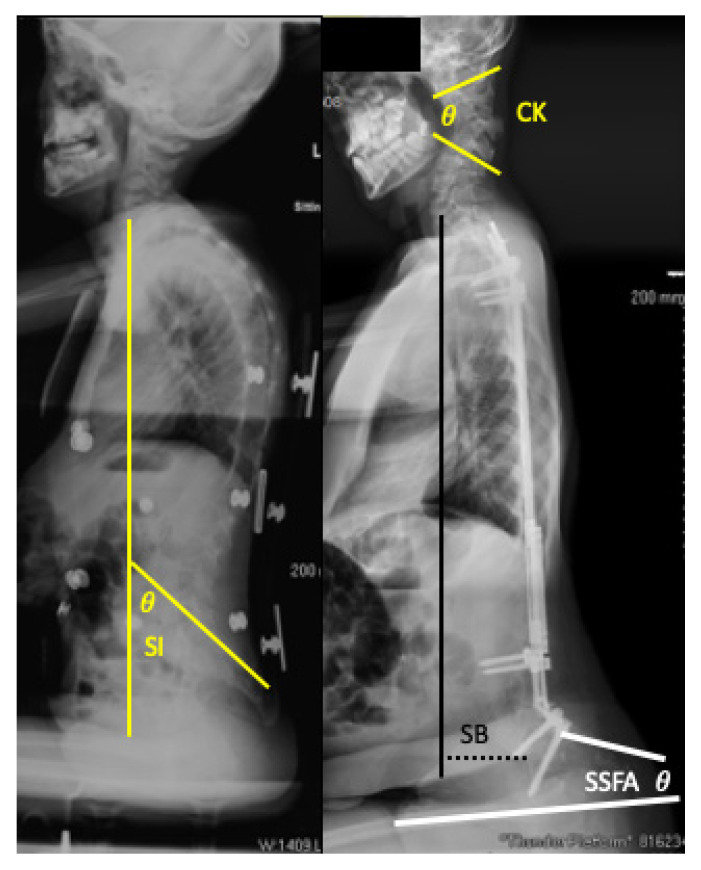
Examples of radiographic sagittal parameters used to objectively characterize deformities. These include Sacral Incidence (SI, Yellow); Cervical Kyphosis (CK, Yellow); Sagittal Balance (SB, Black); Seated Sacral Femoral Angle (SSFA, White).

Prevalence of the deformities was then determined from this data. Demographic comparisons including sex, age at surgery, and post-operative length of follow-up between cohorts was then performed. Being a tertiary referral center for these patients, some of the patients had their procedure performed at outside institutions. As such, exact operative dates were not available for every child, however the year of surgery was able to be deduced from available records. In such instances (4/32) the operative date was assigned to be December thirtieth of the operative year, to assure we were not over-estimating follow-up. To objectively characterize the deformities, “tucked chin” deformities were assessed by cervical sagittal Cobb angles and apex of deformity, while “tipped trunk” deformities were assessed by sagittal balance (C7 plumb line distance to anterior S1 endplate), Sacral Inclination (SI), and Seated Sacral Femoral Angle (SSFA) (a new measurement defined by a line tangential to the posterior sacrum and a line parallel with the anterior femoral shaft) (Figure 3). These values were then compared between those identified with and without the deformities in our screening. The same radiographic measurements described above were then performed on available pre-operative and post-operative radiographs, to assess whether these deformities were present pre-operatively, appeared in post-operative period as a result of surgery, or developed throughout the follow-up period. Finally, pelvic obliquity, coronal deformity, thoracic and lumbar sagittal Cobb angles, instrumentation levels, and hip status (reduced, subluxated, dislocated) were assessed between those with and without the obvious sagittal deformities to identify factors associated with the development of these deformities. All radiographic measurements were made using digital radiographs and measurement tools available through our clinical picture archiving and communicating system (PACS) (McKesson, San Francisco, CA, USA).

Statistical analysis to compare the radiographic variables between those with deformity and those without was performed using an unpaired Student *T*-Tests. All categorical variables were assessed using Fisher’s Exact test. Significance for all statistical comparisons were defined as *p* < 0.05.

## 3. Results

Prevalence of Obvious Deformities in our Population: Thirty-two patients with SMA type II with a history of either spinal fusion (N = 20) or growing rods (N = 12), performed between 1993 and 2015, were identified with an average of 7.6 (2.1–16.6) years post-operative follow-up. Latest lateral radiographs for each of the patients were used to grade the deformities. Obvious “tucked chin” (cervical kyphosis (N = 4)), “tipped trunk” (N = 9), or both (N = 3) deformities resulted in a total of 13/32 (40%) of the patients were identified as having a deformity, the breakdown of the scoring of the 32 can be found in Table 1. Those with deformities had significantly longer follow-up (10 (3.2–16.6) years versus 5.9 (2.1–14.7) years; *p* < 0.01) than those that did not (Table 2). No significant differences were found in the presence of the deformities between those with spinal fusion versus those with growing rods (*p* = 0.76).

Radiographic Characterization of Deformities: Of the 32 patients, 26 had upright follow-up radiographs available for review. As these deformities fall outside the region of instrumentation, not all measurements could be made on every film as some films were focused only on the instrumented levels (Table 3). Cervical kyphosis was greater at final follow-up (53° (37–61°) vs. −24° (−70–11°), *p* < 0.001) in those identified with a tucked chin, with the kyphotic apices in these four being located at C1–2, C2–3,C4,C5; much more proximal than classic proximal junctional kyphosis. Those identified with an obvious tipped trunk demonstrated a more positive sagittal balance (63 mm (0–165 mm) vs. 16.4 mm (−48–59 mm), *p* = 0.04) and an increased anterior tilt of the entire pelvis (demonstrated by the increased sacral inclination (SI) 74.1° (60–94°) vs. 46.8° (32–66°), *p* < 0.0001) and the decreased seated sacral femoral angle (SSFA) (2° (−13.9–8.2°) vs. 35° (8–58°), *p* < 0.0001) (Table 4).

Temporal Appearance of Deformities: The lack of standard adequate pre-operative radiographs limits interpretation of the pre-operative status of the deformities, however, no significant differences were found in mean cervical kyphosis, sagittal balance, SSFA between cohorts. Immediate post-operative radiographs also failed to demonstrate a difference between cohorts in these measurements. Post-operative SI was the only measurement found to be significantly different greater 69° (52–88°) vs. 55° (28–77°) *p* = 0.03, in those with an ultimate tucked chin or tipped trunk deformity (Table 4).

Variables Contributing to the Deformities: No differences in any of the pre-operative Cobb angles or immediate post-operative coronal or lumbosacral Cobb angles were identified over the instrumented segments between groups (Table 5).

The lack of adequate standardized radiographs severely limits the interpretation of the preoperative data. Interestingly, the final thoracic kyphosis (throughout the instrumented levels) was significantly less in those that developed a subsequent sagittal deformity 24° (−12–46°) than in those that did not (45° (9–87°) *p* = 0.008; while lumbar lordosis was the same (Table 6). This resulted in a significantly greater overall lordotic imbalance (Defined as a sum of thoracic kyphosis-lumbar lordosis) throughout the instrumented levels in the spines that developed subsequent deformity compared to those that did not (−30° (−70°–(−)0.3°) vs. −6° (−33.9°–52.8°), *p* = 0.001). Visual inspection of residual plots produced from the linear mixed effects analysis (performed to determine statistical differences in sagittal measurements over time) failed to reveal any obvious deviations from homoscedasticity or normality and indicated a significant effect of thoracic kyphosis on the presence of deformity. Descriptive analysis did not reveal any obvious differences in the levels of instrumentation (Table 7) or in hip status (Table 8).

## 4. Discussion

Scoliosis is common in all types of spinal muscular atrophy (up to 92% of patients with type 1 and 2 and 50% Type 3) and spinal deformities occur earlier with increased disease severity (Type 1 < 2 years of age, Type 2: 1–7 years of age, Type 3: 4–14 years of age) [12,13]. Posterior instrumented fusions [14,15,16,17] or distraction-based growing systems [10,11,18] have been recommended for progressive scoliotic curves in the 50–60 degree range [19]. Due to the relatively rare nature of the disease, most previous studies have grouped sub-types of SMA patients [13,16,20] or included other neuromuscular diagnoses in their analyses and reports [21,22,23,24]. These studies have focused on determining if such procedures were safe and effective [14,23] and how they affected pulmonary status [11,20,25,26], patient function and satisfaction [24,27]. While the effects of early fusion on coronal curve progression have been reported [17], no studies to date have described the effects that spinal stabilization has on the sagittal alignment above or below the instrumented levels in children with SMA.

**Figure 4:**
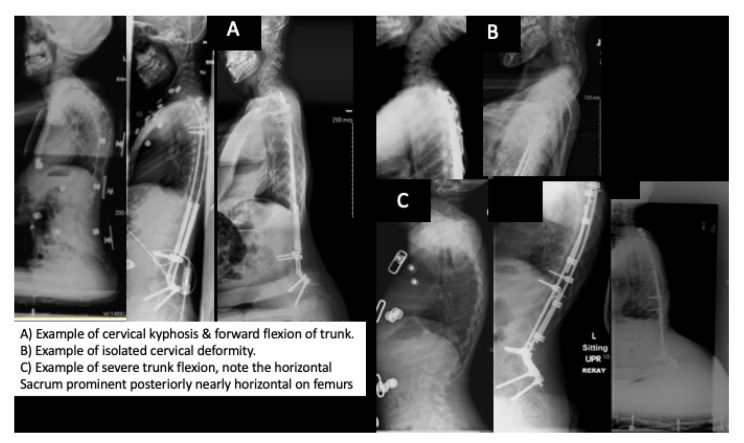
Examples of the cervical (**A**,**B**) and trunk deformities (**A**,**C**) developing over time.

In this study, we describe obvious deformities which occur in the sagittal plane of children with SMA type 2, above and below previously instrumented segments. For years, we have noticed these clinical deformities in our SMA population, however for the most part they have only caused seating issues, especially in those with a tipped trunk as the prominence of the buttock makes spine support difficult (Figure 1). Prior to this work, we had presumed that the children with the tipped trunk were either instrumented in excessive lordosis or perhaps there had been a gradual increase in lumbar lordosis with either continued growth or subtle loss of pelvic fixation given the known low bone density in these children [28,29,30,31,32]. However, after one child in our cohort required surgical treatment for severe cervical kyphosis with neurologic symptoms, we set out to critically assess how many others had such sagittal plane deformities: looking above and below the instrumented levels. In doing so, we demonstrated that these deformities are relatively common and that they are not associated with changes in lumbar lordosis (Table 6 and Figure 4).

Similar changes in sagittal alignment have been described cephalad [33,34,35,36,37,38,39] and/or caudal [40,41,42] to posterior instrumentation in children with adolescent idiopathic scoliosis. However, we are not aware of any other reports describing these changes we have observed in the SMA population. Interestingly, the opposite cervical deformity (hyperextension) has been reported following posterior spinal fusion in children with Duchenne Muscular Dystrophy [43].

From our data, it appears, that children with SMA type 2 are sensitive to hypo-kyphosis (or excessive overall relative lordosis (subtracting the lumbar lordosis from the thoracic kyphosis) following spinal instrumentation. Interestingly, little difference in kyphosis was found between cohorts immediately after surgery, but rather, that kyphosis lessened over time in those with a deformity yet increased in those without a deformity. As the cohort that developed these deformities had significantly longer follow-up, time will tell if more of these deformities develop in the remainder of these patients. As thoracic kyphosis lessened over time in the deformity group, these findings suggest that there may have been subtle anterior growth or crankshafting following the initial posterior spinal instrumentation (Figure 4). Why the average kyphosis increased over time in those without a deformity and why certain children developed cervical deformities and others trunk deformities may not be as easy to answer, as no statistical difference was found in terms of age or instrumentation type (fusion versus growing rods) (Table 3). Perhaps the increase in Sacral Incidence seen immediately post-operative contributes to the likelihood of trunk tip or that subtle preoperative cervical kyphosis or post-operative head positioning contributes to later cervical kyphosis. Lack of adequate upright pre-operative cervical imaging for every patient leaves only conjecture. The authors had hypothesized that the forward tipped trunk may occur more readily in the presence of dislocated hips as the proximal migration of the femurs may act to over lengthen the hamstrings and gluteal muscles allowing the pelvis (and the attached, fused spine) to tip forward in response to the lordotic imbalance, however our limited sample size did not support this explanation.

The lack of uniform, adequate, upright lateral radiographs is the main limitation of this study. While being a tertiary referral center for these children provided the necessary patient volume to allow recognition of the clinical deformities; it complicates assuring that all patients have uniform imaging at their referring institutions and that all images ended up in our PACS for review. This was especially true over the study period as many institutions were transitioning from standard radiographs to digital imaging during this time frame (1993–2015). Furthermore, the underlying diagnosis also complicates standard imaging as their overall weakness and spinal deformities can make upright radiographs for some impossible. Thus, while having full sets of pre-, post- and follow-up radiographs would have strengthened this study, the authors feel that the available radiographs were able to bring to light the ultimate sagittal deformities and highlights to others caring for these children the importance in obtaining AP and lateral upright sitting radiographs including the cervical spine and femurs before and after spinal surgery if possible. Furthermore, while the authors acknowledge that the lack of adequate radiographs severely limited our evaluation into the cause of the deformity, enough imaging was available to determine that at least 40% of our children with SMA type 2 and posterior instrumentation developed these deformities. As this prevalence was determined by taking those with an identified deformity and dividing that number by all the children with SMA type 2 and spinal instrumentation cared for at our institution (regardless of adequate films), additional adequate imaging would have only increased this prevalence, if more deformities had been identified.

Focusing on only SMA type 2 children may also be seen as a limitation of the current study. As children with SMA type 1 are unable to sit upright [44,45,46], and those with type 3 have less muscle weakness [47,48], these findings may be unique to SMA type 2 children. However, as more children are treated with the newer disease modifying drugs [49,50,51,52,53], the classic typing of SMA may become blurred as children become stronger [54,55]. Given the fact that the incidence of type 1 nearly doubles that of type 2 [56,57], we may find many more children with a phenotype similar to the classic SMA type 2 that may require spine surgery and develop compensatory deformities described in this study.

Exactly why these deformities develop and how best to prevent them was not completely answered in this study. It may be the result of a combination of several factors. First, the relative stiffness of the implants may result in a concentration of forces at the cephalad and caudal ends of the implants. Second, the overall muscle weakness from the disease itself results in the lack of muscular support for the unfused segments of the spine. A similar effect has been previously described as it relates to the collapse of the rib cage in children with spinal muscular atrophy known as the parasol rib deformity. [58,59] Finally, the crankshaft effect may develop with the continued growth of the anterior spinal column. Fujak et al. described the crankshaft phenomenon occurring in patients with SMA treated with telescopic rods and recommended definitive spinal fusion between the ages of 10–12 [60]. We have demonstrated safety and overall good results in these patients using standard distraction based growing rods [10,11]. While the authors would not suggest anterior spinal fusion in these children given their underlying pulmonary issues [4,20,61,62,63], surgical variables such as increased frequency of lengthening (using magnetically controlled devices) [64], three-column fixation (pedicle screws) [65,66,67,68] or stiffer instrumentation [69] might provide strategies to prevent the hypokyphosis from occurring. Thus, moving forward, it will be important for the spinal deformity surgeon to be aware of these potential sagittal compensations and to determine the best intervention to prevent them.

One final question that remains unanswered is the potential effect of recent disease modifying therapies on the development of the described sagittal plane deformities. The use of these therapies was not controlled for with this study as most of the study period predated the widespread use of these agents at our institution and could be the focus for future studies.

## 5. Conclusions

This single center, retrospective radiographic analysis demonstrated a 40% prevalence of sagittal deformities occurring above and below posterior instrumentation in SMA type 2 patients and provide radiographic parameters to assess for these deformities. While only correlative, these patients appear very sensitive to a lordotic imbalance that develops following posterior spinal instrumentation resulting in cervical kyphosis or anterior tipping (i.e., flexion) of the trunk. From this study, the authors would recommend all children with SMA being evaluated with scoliosis to have upright radiographs extending from the skull to the femurs, particularly in the lateral view, to allow for the detection of these deformities. Children with significant cervical kyphosis should be evaluated for signs of myelopathy [70], masked by their underlying neurologic pathology. The authors would also recommend caution in the complete correction or over-correction of thoracic kyphosis during spinal instrumentation. Furthermore, while only a correlative risk factor, the continued loss of thoracic kyphosis following instrumentation might be mitigated by (1) increased frequency of growing rod lengthening, (2) stiffer posterior spinal rods and (3) additional points of three-column fixation (pedicle screws), but the authors would caution against each of these interventions as they may have other unintended negative consequences. Further follow-up and studies are necessary to determine the long-term effects of these compensations and to identify strategies to avoid them.

## Figures and Tables

**Table 1:** Results of screening most recent lateral radiographs for evidence of clinically recognized deformities (N = 32).

Deformity	Scoring	Tucked Chin	Trunk Tip
None	0	25	8
Mild	1	3	12
Obvious	2	4	12

**Table 2:** Breakdown of cohorts studied and available radiographs.

	No (Obvious) Deformity	Obvious Deformity	Prevalence
Tucked Chin	0	4	13%
Trunk Tip	0	9	28%
Any	0	13	41%
Both	0	3	9%
Follow-up Radiographs	19	13	
Upright Follow-up Radiographs	14	12	

**Table 3:** Demographic differences between those found with and without obvious deformities on screening.

	No Deformity	Deformity	*p* Value
Number of Type 2 Patients	19	13	
Age at Spinal Surgery	9.2 (4.1–19.1) years	7.9 (4.0–10.7) years	0.3
Fusion	11	9	0.71
Growing Rods	8	4	
Male:Female	7:12	6:7	0.71
Length of Clinical Post-Op Follow-up	5.9 +/− (2.1–10.5) years	10 (3.2–16.6) years	0.002
Length of Post-Op Radiographic Follow-up	5.7 (1.1–11.5) years	8.0 (1.0–16.6) years	0.1

**Table 4:** Sagittal parameters between those identified with and without obvious deformities on screening. Sagittal measurements were only performed on upright radiographs. These data indicate that subjective screening identified objectively measured differences. N = (number with upright lateral that allowed for each measurement/total number with upright radiographs).

Latest Follow-Up	No Deformity		Deformity		*p*-Value
	Average Measure	N with radiographs	Average Measure	N with radiographs	
Tucked Chin					
Cervical Sagittal Cobb (degrees)	(−)24.1 (−69.9–11.2)	(13/14)	52.5 (37.2–61.3)	(4/4 w/cervical deformity)	<0.0001
Trunk Shift					
Sagittal Balance (C7-S1) (mm)	16.4 (-48–59)	(12/14)	62.5 (0–165)	(8/12)	0.04
SI (degrees)	46.8 (32–66)	(14/14)	74.1 (60–94)	(11/12)	<0.0001
SSFA (degrees)	34.7 (7.9–57.7)	(13/14)	2.2 (−13.9–8.2)	(8/12)	<0.0001

**Table 5:** Temporal analysis of sagittal parameters between those that ultimately developed a deformity or did not. Lack of adequate upright radiographs limits interpretation of the data, however, from the available data no significant differences were found.

	Pre-Operative		*p*-Value	Immediate Post-Operative		*p*-Value
**No Deformity (N = 19)**	Average Measure	N with radiographs	No Deformity vs. Deformity	Average Measure	N with radiographs	No Deformity vs. Deformity
Cervical Sagittal Cobb	NA	0	NA	(−)10.5 (−51–28)	17	0.23
Sagittal Balance (C7-S1)	53.9 (15.3–96.4)	8	0.05	14.5 (−47.6–58.6)	19	0.43
SI	32.5 (9–56)	9	0.49	55.2 (28–77)	18	0.03
SSFA	45.5 (20.2–74.7)	8	0.88	39.6 (27–66)	17	0.16
**With Deformity (N = 4/N = 13)**	Average Measure	N with radiographs		Average Measure	N with radiographs	
Cervical Sagittal Cobb (N = 4)	(−)0.9 (−7.8–6.1)	2		8.5 (−57.1–44.1)	4	
Sagittal Balance (C7-S1)	7.4 (−44.3–66.7)	5		(−)2.4 (−50.7–61.4)	4	
SI	39.2 (19–56)	6		66.9 (52–88)	13	
SFA	47.8 (26.7–75.4)	5		28.8 (0–72)	13	

**Table 6:** Analysis of spinal parameters at each time point, comparing those with and without spinal deformities.

	**Pre-Operative**			Immediate Post-Operative			Latest Radiographs *			Only Upright Latest Radiographs		
**No Deformity (N = 19)**	Average Measure	N ^	*p*-Value **	Average Measure	N ^	*p*-Value **	Average Measure	N ^	*p*-Value **	Average Measure	N ^	*p*-Value **
Coronal Cobb	61.8 (30.7–118)	15 *	0.65	37.2 (7.3–85.7)	19	0.14	37.5 (8.1–108)	19 *	0.09	32.3 (2.7–62.6)	13	0.07
Pelvic Obliquity	28.3 (12.3–40.8)	15 *	0.18	11.3 (1–56)	19	0.78	9.8 (0.4–58)	19 *	0.93	9.6 (0.4–58)	13	0.97
Sagittal Cobb T/L (Kyphosis)	72.3 (41–101)	7 *	0.93	37.3 (5–63)	18	0.29	44.7 (8.7–87.4)	19 *	0.008	41.7 (8.7–87.4)	13	0.03
Sagittal Cobb L/S (Lordosis)	49.5 (8.3–82.2)	11 *	0.92	50.6 (14–78)	19	0.36	49.2 (8.0–79.7)	17 *	0.88	47.3 (8.0–79.7)	13	0.85
Kyphosis-Lordosis	26.2 (7.2–71.1)	5 *	0.79	(−)16.1 (−35.6–7.4)	18	0.28	(−)5.6 (−33.9–52.8)	17 *	0.001	(−)5.6 (−33.9–52.8)	13	0.005
**With Deformity (N = 13)**	Average Measure	N ^		Average Measure	N ^		Average Measure	N ^		Average Measure	N ^	
Coronal Cobb	56.2 (39.9–59.9)	6		27.5 (8.6–54.1)	13		21.2 (2.3–63.9)	12 *		17.9 (2.3–63.9)	11	
Pelvic Obliquity	17.6 (6.5–28.5)	6		10.1 (1.2–26.4)	11		9.4 (0.2–46.5)	11		9.4 (0.2–46.5)	11	
Sagittal Cobb T/L (Kyphosis)	73.1 (51.7–94.6)	7		30.1 (−4.8–50)	11		24.2 (−11.8–46.4)	13		24.1 (−11.8–46.4)	13	
Sagittal Cobb L/S (Lordosis)	51 (16.8–77.5)	7		49.8 (37.1–76.1)	11		54.6 (37–74.2)	13		54.6 (28.6–74.2)	13	
Kyphosis-Lordosis	22.2 (−26.9–52.8)	7		(−)21 (−35.6–7.4)	10		(−)30.4 (−70.3–(−)0.3)	13		(−)30.4 (0.3–70.34)	13	

^ N = number with radiographs; * Includes supine films; ** *p*-value = No deformity versus deformity.

**Table 7:** Comparison of instrumentation between those with and without deformities.

		No Deformity	Deformity
Instrumentation		N = 19	N = 13
Proximal	T1	2	0
	T2	12	11
	T3	3	2
	Below	2 *	0
Distal	L4 or L5	7	2
	Pelvis/Sacrum	12	11
	Revision Proximal	2 *	0
	Revision Distal	1 ^	1

* Proximal implants revised. ^ Distal implants removed and later revision.

**Table 8:** Comparison of hip status between those with and without deformities.

	No Deformity	Deformity
Hip Status	N = 19	N = 13
B/L Dislocation	2	2
Unilateral Dislocation	2	0
Uni Dislocation + Uni Subluxation	5	1
B/L Subluxation	4	2
Unilateral Subluxation	3	4
B/L Reduced	0	1
Inadequate Films to Assess	3	3

## Data Availability

Data is contained within the article.
